# The diagnosis of right heart thrombus by focused cardiac ultrasound in a critically ill patient in compensated shock

**DOI:** 10.1186/s13089-015-0023-7

**Published:** 2015-05-13

**Authors:** Mansour Jammal, Peter Milano, Renzo Cardenas, Thomas Mailhot, Diku Mandavia, Phillips Perera

**Affiliations:** Division of Emergency Medicine, 300 Pasteur Drive, Alway Building, M121, Stanford, CA 94305 USA; Department of Emergency Medicine, Los Angeles County + USC Medical Center, 1200 N. State St # 1011, , Los Angeles, CA 90033 USA

**Keywords:** Right heart thrombus, Pulmonary embolus, Echocardiography, Cardiac ultrasound, Focused cardiac ultrasound (FocUS), Bedside ultrasound, RUSH exam-rapid ultrasound in shock exam in the critically ill patient, Ultrasound in shock, Ultrasound in shortness of breath

## Abstract

Right heart thrombus (RHT) is a life-threatening diagnosis that is rarely made in the emergency department (ED), but with the increasing use of focused cardiac ultrasound (FocUS), more of these cases may be identified in a timely fashion. We present a case of an ill-appearing patient who had an immediate change in management due to the visualization of RHT soon after arrival to the ED. The diagnosis was confirmed after a cardiology-performed ultrasound (US). This case illustrates the value of the recognition of RHT on FocUS and how US protocols designed for the evaluation of shock and shortness of breath may potentially be expanded to patients in a ‘compensated’ or ‘pre-shock’ state to expedite the correct diagnosis and to facilitate more timely management.

**Electronic supplementary material:**

The online version of this article (doi:10.1186/s13089-015-0023-7) contains supplementary material, which is available to authorized users.

## Background

Right heart thrombus (RHT) is a challenging diagnosis based solely on clinical presentation. The majority of thrombi originate from the deep venous system and embolize to the right heart as precursors of a pulmonary embolism (PE) [1]. Identification of these ‘thrombi in transit’ is rare and infrequently done in the emergency department (ED). As a result, the associated mortality levels have been reported to be high, ranging from 21% to greater than 40% [2,3]. Much of the difficulty in making this diagnosis is due to the wide spectrum of manifesting features, which include near-syncope, undifferentiated tachycardia, shortness of breath, and shock, with many permutations in-between. The diagnostic modality of choice for suspected pulmonary embolism in the ED is computed tomographic angiography (CTA) [4]. However, it is often complicated to transfer hemodynamically unstable patients to the radiology suite for CT imaging, due to the high potential for acute deterioration. In addition, hemodynamically stable patients with significant kidney disease are not optimal candidates for CTA, due to the risk of contrast nephropathy. Historically, alternative methods of diagnosis that include cardiology-performed transthoracic echocardiography (TTE), transesophageal echocardiography (TEE), and ventilation-perfusion (VQ) scan are difficult to obtain urgently. However, with the expanding use of clinician-performed focused cardiac ultrasound (FocUS), the ability to identify a free-floating RHT can be expedited and may drastically decrease the time to diagnosis and appropriate management, thus potentially improving a patient’s morbidity and mortality. In this case report, we describe how focused FocUS rapidly diagnosed RHT and immediately changed therapeutic direction.

## Case presentation

### A) Initial presentation

A 50-year-old man with history of cellulitis and amphetamine abuse was brought to the ED for the evaluation of bilateral leg pain. He described a week of progressive lower extremity edema, shortness of breath, fevers, chills, night sweats, and worsening lower extremity pain. He stated that the pain was so severe that he could not ambulate and was effectively bedbound.

On physical examination, he was pale and ill-appearing and was complaining of bilateral leg pain. His oral temperature was 97.5°F, heart rate was 150 beats per minute, blood pressure was 101/56 mm Hg, respiratory rate was 16 breaths per minute, and oxygen saturation was 100% on room air. He was alert to name and place, but slow to respond. Cardiac examination was remarkable for tachycardia without murmur, and no jugular venous distention was present. His pulmonary exam revealed lungs that were clear to auscultation bilaterally. Examination of his lower extremities demonstrated bilateral pitting edema to the thighs, right greater than left, with erythema and multiple weeping erythematous plaques over the skin.

**Figure 1:**
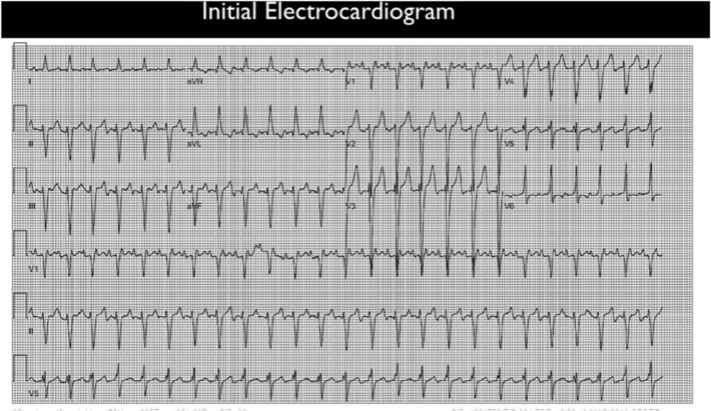
EKG demonstrated rapid arrhythmia with atrial flutter conducting at a 2:1 block.

**Figure 2:**
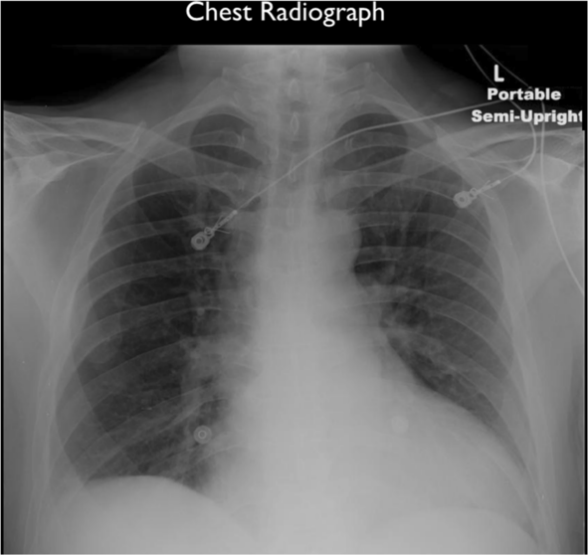
CXR showed an enlarged heart without acute infiltrate, effusion, edema or pneumothorax.

Electrocardiogram (EKG) demonstrated atrial flutter conducting with a 2:1 block (Figure 1). Chest radiograph showed an enlarged heart without acute infiltrate, effusion, edema, or pneumothorax (Figure 2). Laboratory studies were significant for an elevated white blood count of 21,800/mm^3^ with 89% neutrophils, venous lactate 2.9 mmol/L (normal <2.2 mmol/L), sodium 121 mmol/L, potassium 5.8 mmol/L, chloride 86 mmol/L, bicarbonate 19 mmol/L, blood urea nitrogen 47 mg/dL, creatinine 2.0 mg/dL, and urine toxicology screen positive for amphetamines.

**Figure 3:**
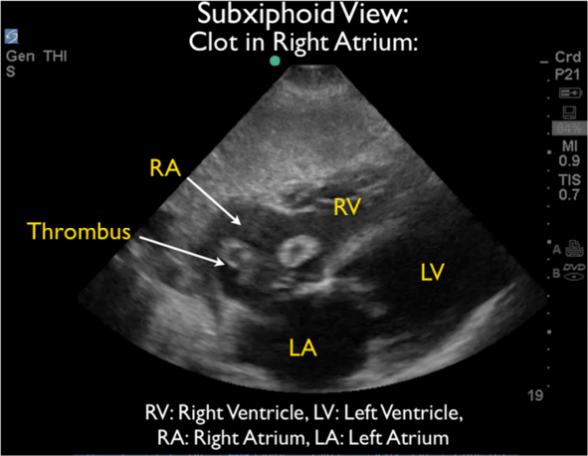
Subxiphoid cardiac echo view demonstrated thrombi within the right atrium. LA, left atrium; LV, left ventricle; RA, right atrium; RV, right ventricle.

**Figure 4:**
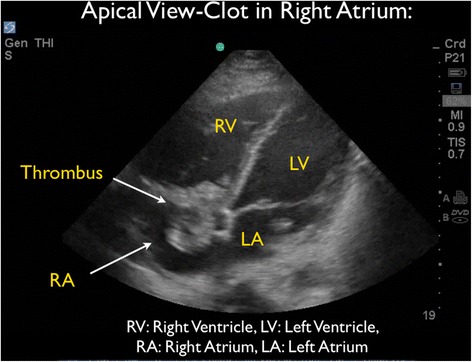
Apical cardiac echo view demonstrated thrombi within the right atrium. At times thrombi were seen moving into the right ventricle through the tricuspid valve. LA, left atrium; LV, left ventricle; RA, right atrium; RV, right ventricle.

**Figure 5:**
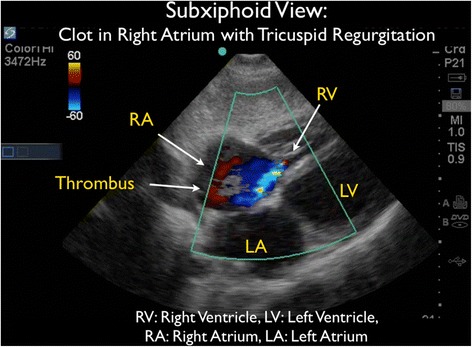
Doppler US interrogation of the tricuspid valve taken from the subxiphoid echo view. Significant tricuspid regurgitation is present and this blood flow moves the thrombi retrograde into the right atrium from the right ventricle. LA, left atrium; LV, left ventricle; RA, right atrium; RV, right ventricle.

**Figure 6:**
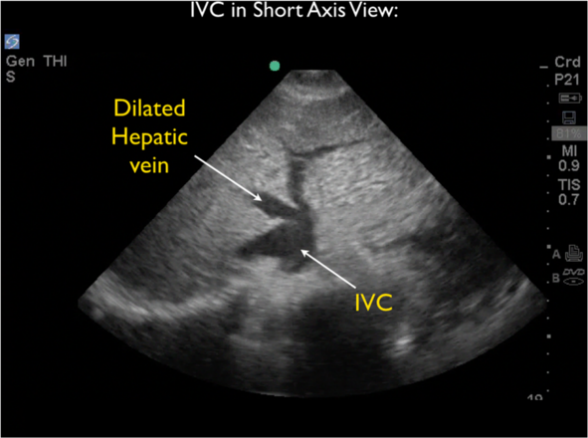
The inferior vena cava (IVC) was evaluated in a short-axis view. The IVC was distended with little respiratory variation, indicating a relatively elevated central venous pressure (CVP).

**Figure 7:**
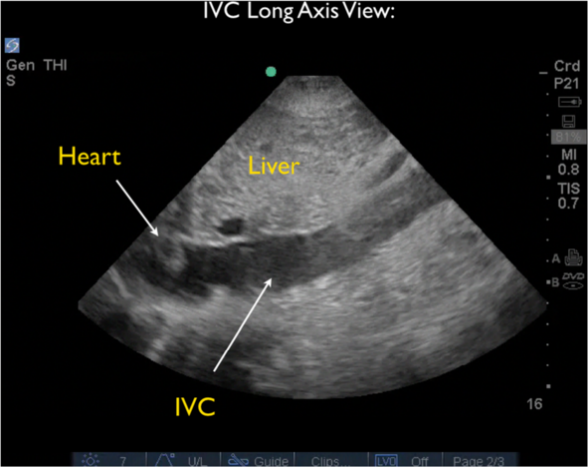
The inferior vena cava (IVC) was evaluated in a long-axis view. The IVC was distended with little respiratory variation, indicating a relatively elevated central venous pressure (CVP).

As the patient appeared to be critically ill with significant tachycardia, FocUS was immediately performed and demonstrated very poor left ventricular contractility, a small pericardial effusion, and moderate enlargement of the right ventricle. A series of thrombi were clearly visualized, swirling within the right atrium (RA). At times, the thrombi were seen to move back and forth across the tricuspid valve (TV) (Figures 3 and 4 and Additional files 1 and 2). Interestingly, on Doppler ultrasound (US) interrogation of the TV, the patient was noted to have severe tricuspid regurgitation (TR). The significant TR jet was observed to move the thrombi retrograde from the right ventricle (RV) back into the RA, effectively preventing forward flow of the clots into the pulmonary artery (Figure 5, Additional file 3). Next, the inferior vena cava (IVC) was evaluated in two planes, using both short- and long-axis views. The IVC was distended with little respiratory variation, findings more consistent with a relatively elevated central venous pressure (CVP) (Figures 6 and 7 and Additional files 4 and 5).

Due to the FocUS findings suggesting poor cardiac function and relatively elevated CVP, the initial order for a bolus with intravenous fluid was canceled. A diltiazem drip was then administered to further slow the ventricular rate. Unfractionated heparin was started for treatment of the atrial masses, presumed to be thrombi. Cardiology, pulmonary/critical care medicine and interventional radiology were consulted regarding therapeutic options. Administration of tissue plasminogen activator (t-PA), as well as catheter-based removal of the thrombi, were discussed. However, due to the patient’s poor medical condition, these more aggressive options were deferred. He was then admitted to the intensive care unit (ICU) with the diagnoses of RHT, atrial flutter, cardiogenic shock, sepsis, and acute renal failure.

### B) Hospital course

On arrival to the ICU, the attending cardiologist consulting for the ICU performed a bedside US. This documented ‘confirmed presence of a RA abnormality, that is more suspicious for a vegetation, it is unclear whether this is attached to the atrial wall or to the TV’. Gentamycin was added to broaden antibiotic coverage, due to concern for endocarditis. An amiodarone drip was started for atrial flutter, with subsequent conversion to sinus rhythm.

On hospital day #2, a comprehensive cardiology TTE showed the following findings: a large, freely mobile, largely cylindrical echodensity in the RA, measuring approximately 8 cm in length (stretched out) and 1.4 cm in diameter, suspicious for thrombus. Throughout the cardiac cycle, the mass predominantly rotated freely within the RA, but at different times, portions of this mass was seen both entering into and exiting from the IVC and the RV. Also reported was mildly dilated left ventricular size and global hypokinesis. The estimated ejection fraction was severely reduced at <20%. Moderately dilated RV size was also appreciated, with reduced systolic function. The RA was significantly dilated. The inter-atrial septum was noted to bow toward the left atrium, consistent with abnormally elevated RA pressures. The TV leaflets were apically tented, with moderate-to-severe TR. The RV systolic pressure was estimated to be elevated at 40 to 45 mmHg.

Cardiothoracic surgery was consulted due to these TTE findings, but deemed the patient to be a poor surgical candidate for operative removal of the thrombi. Heparin was continued, and thrombolytics were kept at the bedside ready for potential use. A bilateral lower extremity Doppler US demonstrated no evidence of deep vein thrombosis (DVT) from the femoral vein to the popliteal vein. The patient’s renal failure resolved with normal saline administered slowly over several days.

On hospital day #4, presumably based on the TTE reading favoring a thrombus over vegetation, the cardiology service documented the following consultation: ‘There is no safe mechanism by which atrial thrombus can be removed in this patient, and we recommend continued treatment with heparin’. The patient subsequently had continued clinical improvement that allowed for transfer from the ICU to the medicine floor while receiving vancomycin, piperacillin/tazobactam, heparin, and oral amiodarone. Blood and urine cultures drawn prior to the administration of antibiotics were negative for growth at 5 days.

**Figure 8:**
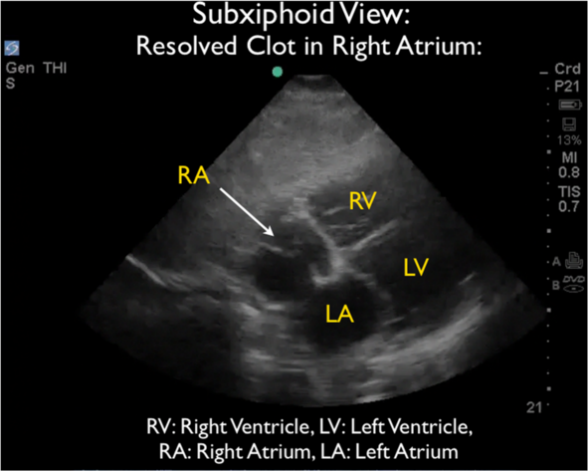
A repeat bedside US taken from the subxiphoid view on the day of discharge. Demonstrated resolution of thrombi in RA. LA, left atrium; LV, left ventricle; RA, right atrium; RV, right ventricle.

**Figure 9:**
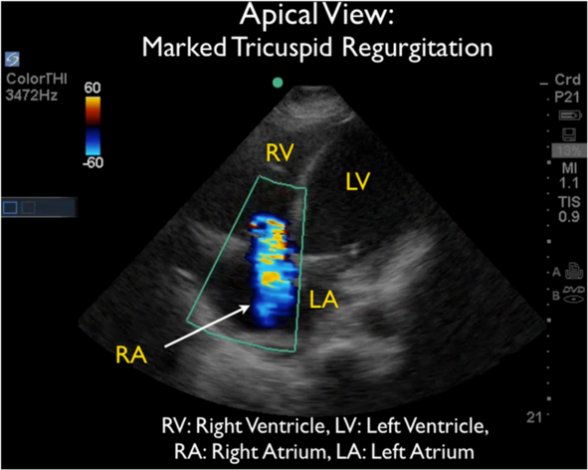
A repeat bedside US taken from the apical view on the day of discharge. Demonstrated the continued presence of tricuspid regurgitation. LA, left atrium; LV, left ventricle; RA, right atrium; RV, right ventricle.

On hospital day #9, he had been transitioned to oral warfarin and was discharged home. On the day of discharge, a repeat FocUS performed by the attending emergency physician who initially cared for the patient demonstrated resolution of RA thrombus with persistence of significant TR (Figures 8 and 9 and Additional files 6 and 7). As the patient had significant clinical improvement on heparin therapy, no further studies such as CT angiography or TEE were obtained during the hospital course.

### Case discussion

The case describes a complex case of RHT. Because the patient was ill-appearing with significant tachycardia and an abnormal shock index (HR 150 beats per minute/BP 101 mm Hg = 1.5, abnormal >1) upon arrival to the ED, FocUS was performed in this patient who manifested a ‘compensated shock state’. Interestingly, sepsis with arrhythmia was the initial physician diagnostic impression, rather than thromboembolic disease as the Wells score for the patient was calculated to be 6, putting him only into the moderate risk category for PE (bilateral swelling of the legs, heart rate greater than 100 beats per minute). However, the FocUS examination on initial evaluation revealed the mobile RHT, which immediately facilitated the correct diagnosis and ultimately changed the patient’s entire medical management throughout his hospitalization.

While the rapid ultrasound in shock in the critically ill (RUSH) exam was the ultrasound protocol employed in this patient, this represents one of many US examinations that have been developed for use in the patient with hypotension and/or shortness of breath [5-31]. A recent study found the RUSH exam to have good sensitivity and positive predictive value in defining the type of shock in patients presenting with undifferentiated hypotension [32]. Due to the acuity of the patient’s presentation, an abbreviated RUSH exam that specifically included cardiac and IVC US exams was used in this case. Lung examination (specifically, looking for alveolar interstitial syndrome and pleural effusions) may have added very useful information in this case and was only deferred secondary to the patient’s severity of illness and difficulty cooperating with a full exam.

In patients with a diagnosed pulmonary embolism, RHT is uncommon, ranging from 3.6% to 23.3% of cases [2,3,33-36]. The publication with the highest rate of RHT [35] enrolled only patients with a diagnosis of massive PE (all either presented with cardiogenic shock or severe shortness of breath), which likely led to the increased incidence as compared to other studies. Visualized RHT has also been described as a potential cause of cardiac arrest in a patient who was found to have bilateral and massive PE on CT scan after the return of spontaneous circulation [37].

The two major etiologies of RHT are embolic (due to propagation of a DVT) or *in situ* (due to stagnant blood flow, as seen in cardiomyopathy and in arrhythmia, such as in atrial fibrillation). However, there are case reports documenting RHT secondary to pacemaker wires [38], central venous catheters [39], hemodialysis catheters [40], and embryological remnants [41], as a complication of ablation [42], formed during cardiac arrest with cardiopulmonary resuscitation [43], and after carbon monoxide poisoning [44]. With regard to our patient, it is likely that the RHT development was multifactorial. He presented with leg pain and swelling in the setting of prolonged immobility, suggesting the formation of a lower extremity DVT. Although DVT was not detected on US, it is possible that the thrombus may have already embolized to the heart. This US also checked for DVT in the legs only inferiorly to the level of trifurcation in the popliteal fossa, and a DVT in the calf veins may have potentially been missed. Furthermore, the patient presented with atrial flutter, which often coexists with periods of atrial fibrillation, and predisposes to a higher risk of cardiac emboli than in the general population [45].

RHT morphology has been separated into three types based on results from a multicenter questionnaire on echocardiographically detected RHT [1]: type A - thin, highly mobile, serpiginous; type B - immobile and ovoid shaped; type C - had characteristics of both A and B (highly mobile but globular). Type A thrombi were mostly associated with DVTs, type B thrombi were mostly associated with low-flow states, and type C thrombi included a combination of both. The varying morphologies carried a substantially different prognosis - type A thrombi had a 42% thrombus-related early mortality rate (<8 days after diagnosis), whereas type B and C had thrombus-related early mortality rates of 4% and 14%, respectively. By the appearance of our patient’s initial FocUS, he most likely had a type A thrombus, which portends the poorest prognosis. Thus, rapid detection via FocUS and subsequent facilitated management of the RHT were critical in this case.

Occasionally, intracardiac thrombus may be confused with endocarditis on FocUS. These conditions can be discriminated by the fact that endocarditis is diagnosed as an irregularity on a valvular surface due to a tethered vegetation versus a RHT which is more often freely circulating within a cardiac chamber. However, it may be difficult to visualize this difference on FocUS. In fact, this patient’s initial treatment regimen was broadened to include gentamycin, as the attending cardiologist in the ICU initially believed that the echogenic RA abnormality represented a vegetation on FocUS. The interpretation was later revised when the comprehensive cardiology TTE was performed, which confirmed the presence of thrombus. However, due to the fact that the in-hospital mortality rate for infective endocarditis (IE) is 18% and that there is a direct correlation between size of vegetation and mortality [46], it is important to keep this in mind when an irregular cardiac mass is identified with FocUS.

This case highlights consideration of expanding current shock resuscitation US protocols for use in cases of ‘compensated shock’, especially in patients with undifferentiated tachycardia and an abnormal shock index. Patients in this ‘pre-shock’ state may maintain relatively normal blood pressure measurements, until a tipping point is reached and rapid hemodynamic deterioration ensues. As described prior, the majority of shock US protocols begin with FocUS, looking specifically for the presence of a significant pericardial effusion, the state of left ventricular contractility and for acute dilation of the right ventricle. While the determination of the presence of acute right ventricular dilation is relatively specific for the presence of PE (87% to 98%), it is not a very sensitive finding (31% to 72%) [47]. Addition of US of the lungs and leg veins to FocUS may improve this sensitivity and specificity [47]. The presence of right ventricular dilation indicates relatively high right side heart pressures in relation to the left; however, this may be seen in both acute and chronic conditions. In acute right ventricular dilation, the ventricular wall will generally be thinner due to the lack of compensatory hypertrophy, measuring less than 5 mm at diastole [48,49]. In contrast, in chronic right ventricular strain, such as may exist in patients with primary pulmonary hypertension or chronic obstructive pulmonary disease (COPD), the ventricular wall will be thicker, typically measuring greater than 5 mm. Prominence of the tricuspid valve papillary muscles may also be seen. In fact, in many patients with chronic cor pulmonale, diastolic right ventricular thickness averages 10 mm [50].

Expanding FocUS to look for mobile cardiac RHT may therefore identify additional patients at risk for PE. However, this proposed application of FocUS should currently be considered an advanced application, and should generally be confirmed with a follow-up comprehensive TTE or CT imaging. A further study can identify certain idiosyncrasies related to each type of thrombus, e.g., a patent foramen ovale (PFO) that would necessitate surgical treatment, or a myxoma that may masquerade as a thromboembolus.

The second part of the US examination looked specifically at the IVC to assist in determination of the CVP. In this case, the IVC was plethoric with little respiratory change, indicating a relatively elevated CVP. The initial bolus of crystalloid was therefore deferred. While there is controversy regarding the use of US of the IVC to accurately determine volume status, measurements consistent with the extremes of volume status (like the elevated CVP in this case) are believed to be more reliable [51].

Smoke-like swirling of echogenic material, referred to as spontaneous echo contrast (SEC), was seen within the IVC, although actual thrombus was not seen. SEC in the left atrium has been linked to increased stroke risk, and when seen in the right heart, SEC has been correlated with increased risk of pulmonary embolism [52,53]. The significance of SEC in the IVC is less clear, and echogenic material within the IVC may be seen in a range of patients using newer US machines with improved imaging. One case report described SEC in constrictive pericarditis [54], while another study of 100 patients failed to find any clinical implication of this finding [55]. In one case study, actual thrombus was seen within the IVC moving toward the heart [37]. Therefore, the IVC should be carefully examined during FocUS, not only to determine CVP but also to evaluate for potential thrombus in transit, which may indicate a higher risk of PE. The presence of SEC in the IVC coupled with hemodynamic instability and an enlarged right heart may also suggest the presence of acute right heart obstruction. Further investigation into the significance of SEC in the IVC is warranted.

Our patient had several features in addition to RHT that increased the risk of adverse outcomes. The PE severity index (PESI) was 120, placing our patient in a high-risk (class IV) subset of patients with a 30-day mortality between 4% and 11.4% [56]. Although our patient was not hypotensive, he was hemodynamically unstable and had an elevated shock index, which has been associated with higher in-hospital mortality in patients with PE [57]. Troponin and BNP levels were not sent, but elevations of these biomarkers in the setting of PE has been associated with poor outcomes [58-60]. More recently, an article by Vanni et al. found an increased risk of PE-related complications in patients with elevated lactate levels, such as in this patient [61]. Echocardiographic findings of right heart dysfunction may predict mortality in normotensive patients. In our patient, we detected enlargement of the right ventricle and significant tricuspid regurgitation; however, we did not assess for right ventricular hypokinesis or pulmonary hypertension. These markers of right heart dysfunction in the setting of pulmonary embolism portend a worse prognosis [62-64].

Previously published case reports describe successful management of mobile RHT via anticoagulation with heparin [65-67], systemic thrombolysis [68-74], catheter-directed thrombolysis [75], surgical embolectomy under cardiopulmonary bypass [76-78], and percutaneous embolectomy [79-82]. No consensus has been achieved, as there have been no prospective, randomized trials to date. Thus, the available literature is rife with case reports and retrospective analyses of hospital registries. The case reports present an obvious reporting bias, as instances of failed therapy are less likely to be published. The registries evaluated the prevalence of RHT among patients with diagnosed PE and calculated mortality rates based on treatments that were decided upon by the discretion of the patient’s physicians. Using the ICOPER registry, Torbicki et al. specifically analyzed mobile RHT and found heparin to have a statistically significant inferiority as a sole treatment option (23.5% 14-day mortality for PE with RHT versus 8.0% 14-day mortality for PE without RHT) [33]. However, 14-day mortality rates of thrombolysis versus surgical embolectomy (which included catheter embolectomy) were equivocal - 20.8% versus 25%. In other studies, mortality rates were lowest for those treated with thrombolytics [2,35,83]. In the Ferrari study, there were 18 patients found to have RHT out of 343 diagnosed PE’s [34]. All patients survived, and 16 of them received thrombolytics without any major adverse effects. It is important to remember that selection bias clouds the strength of these studies, as patients that are sicker, i.e., hemodynamically unstable, received more aggressive therapy. It is difficult to determine if these patients had higher mortality rates because of their initial presentation, or due to the inferior efficacy of the treatment itself.

Two prospective trials have been published that enrolled consecutive patients analyzing mobile RHT in diagnosed massive PE. Greco et al. found 7 of 30 (23.3%) patients to have mobile RHT, all of which were successfully treated with thrombolytics without any documented adverse effects [36]. All patients demonstrated lysis of clots between 45 and 60 min of initiation of thrombolytics. In Pierre-Justin and Pierard’s study, RHT was seen in 12 of 335 (3.6%) patients diagnosed with massive PE [84]. Five patients initially were treated with heparin (three of which had contraindications to thrombolysis - all died; the other two received thrombolysis after no improvement with heparin and survived without adverse outcome). The other seven were initially treated with thrombolysis (five improved and two required surgical embolectomy after no improvement with thrombolysis - one died). Both studies concluded that thrombolysis is rapidly effective for the treatment of mobile RHT in the right clinical scenario.

Rose et al. published the only known meta-analysis of the treatment of RHT [77]. A literature search revealed 177 patients with RHT, with the overall mortality rate of 27.1%. When further analyzed, the mortality rates associated with the following treatments were: no treatment (100%), anticoagulation therapy (28.6%), surgical embolectomy (23.8%), and thrombolysis (11.3%). Thrombolytics were considered to have a statistically significant protective effect compared to anticoagulation and surgical embolectomy.

Some patients may be better suited for one therapy over another based on the clinical scenario. Patients presenting in shock may benefit the most from thrombolysis [85]. In contrast, patients who are elderly, who have undergone recent major surgery, and those with metastatic cancer are poor candidates for thrombolysis. In addition, thrombolysis in patients with PFO increases the risk of fragmentation and paradoxical embolus. There have also been case reports of patients who deteriorated after thrombolysis [72,86] with the suspected mechanism being increased clot burden traveling into the pulmonary vasculature after lysis of thrombus. On the other hand, opting for surgery is not without its risks - waiting time to set up the operating rooms, lack of surgical expertise, anesthesia, cardioplegia, and inability to remove or dissolve distal thrombi. In the instances where both thrombolysis and surgery are deemed too high-risk, percutaneous thrombectomy or anticoagulation should be considered. In the case of our patient, the treating physicians felt it too dangerous for him to undergo embolectomy or thrombolysis. He received the more conservative therapy with anticoagulation alone and subsequently improved, despite evidence that heparin by itself is a less effective treatment option as compared to the others.

This patient also had the finding of significant valvular TR on Doppler US, which may have been protective in this case by moving clots that had migrated into the RV back across the tricuspid valve into the RA thus preventing dissemination of the thrombi forward into the pulmonary vasculature. In effect, it is possible that ‘he may have been saved’ by his significant TR.

## Conclusions

This case report underlies the importance of FocUS in the initial evaluation of the undifferentiated critically ill patient. Though many shock US protocols, like the RUSH exam in this case, are now currently used for the evaluation of unexplained hypotension, consideration should be made to also expand these shock US applications to include patients with compensated shock who manifest tachycardia without hypotension and/or an abnormal shock index. Shock US protocols can be expanded to include examination for thrombus in the right heart and IVC as identification of such thrombi in these locations may change immediate management. FocUS should therefore be considered an essential tool for physicians caring for the critical patient, especially in the immediate resuscitation period and in time of sudden hemodynamic deterioration. However, the finding of significant pathology using an advanced application of clinician performed FocUS should generally be followed up with additional testing, such as CTA, V/Q scan, comprehensive TTE, and/or TEE.

## Consent

Written informed consent was obtained from the patient for publication of this case report and any accompanying images. A copy of the written consent is available for review by the Editor-in-Chief of this journal.

## Additional files

Additional file 1:
**Subxiphoid cardiac echo video demonstrating thrombi swirling within the right atrium.**
Additional file 2:
**Apical cardiac echo video demonstrating thrombi swirling within the right atrium, at times moving into the right ventricle through the tricuspid valve.**
Additional file 3:
**Doppler US interrogation of the tricuspid valve taken from the subxiphoid echo view.** Significant tricuspid regurgitation is present, and this jet of blood moves the thrombi back into the right atrium from the right ventricle. Additional file 4:
**The inferior vena cava (IVC) was evaluated in a short-axis view.** The IVC was distended with little respiratory variation, indicating a relatively elevated central venous pressure (CVP). Additional file 5:
**The inferior vena cava (IVC) was evaluated in a long-axis view.** The IVC was distended with little respiratory variation, indicating a relatively elevated central venous pressure (CVP). Additional file 6:
**A repeat bedside US taken from the subxiphoid view on the day of discharge demonstrated resolution of thrombi in RA.**
Additional file 7:
**A repeat bedside US taken from the apical view on the day of discharge demonstrated the continued presence of tricuspid regurgitation.**

## Abbreviations

COPD: chronic obstructive pulmonary disease
CTA: computed tomography angiography
CVP: central venous pressure
CXR: chest radiograph
DVT: deep venous thrombosis
ED: emergency department
EKG: electrocardiogram
FocUS: focused cardiac ultrasound
ICU: intensive care unit
IE: infective endocarditis
IVC: inferior vena cava
PE: pulmonary embolism
PESI: pulmonary embolism severity index
RA: right atrium
RHT: right heart thrombus
RUSH exam: rapid ultrasound in shock in the critically ill
RV: right ventricle
TEE: transesophageal echocardiography
TR: tricuspid regurgitation
TTE: trans-thoracic echocardiography
US: ultrasound
VQ scan: ventilation-perfusion scan.
